# Willingness to receive the second booster of COVID-19 vaccine among older adults with cancer: a stratified analysis in four provinces of China

**DOI:** 10.3389/fpubh.2024.1298070

**Published:** 2024-02-22

**Authors:** Liangyuan Zhang, Jianzhou Yang, Rila Su, Xinquan Lan, Moxin Song, Lei Zhang, Junjie Xu

**Affiliations:** ^1^Clinical Research Academy, Peking University Shenzhen Hospital, Shenzhen, China; ^2^Department of Epidemiology, China Medical University, Shenyang, Liaoning, China; ^3^Department of Public Health and Preventive Medicine, Changzhi Medical College, Changzhi, Shanxi, China; ^4^Cancer Center of Inner Mongolia People's Hospital, Hohhot, Inner Mongolia, China; ^5^Johns Hopkins Bloomberg School of Public Health, Baltimore, MD, United States; ^6^Department of Oncology, Peking University Shenzhen Hospital, Shenzhen, China

**Keywords:** vaccine willingness, second booster of COVID-19 vaccine, cancer, older adults, hesitancy

## Abstract

**Background:**

Despite the elevated COVID-19 risk for older adults with cancer, vaccine hesitancy poses a significant barrier to their immunization. Intriguingly, there is limited research on the prevalence of willingness to receive the second booster dose and associated determinants in older adults with cancer.

**Objective:**

Our objective was to ascertain the level of awareness about COVID-19 vaccines and to uncover the factors influencing the willingness to receive the second booster among Chinese cancer patients aged 65 years and over.

**Methods:**

To achieve our objective, we conducted a multicenter cross-sectional study in four tertiary hospitals from four provinces of China. This involved using a Health Belief Model (HBM) based self-administered questionnaire and medical records. Subsequently, we employed multivariable logistic regression to identify factors influencing the second COVID-19 booster vaccine willingness.

**Results:**

Our results showed that among 893 eligible participants, 279 (31.24%) were aged 65 years and over, and 614 (68.76%) were younger. Interestingly, the willingness to receive the second COVID-19 booster vaccine was 34.1% (95/279) (OR: 1.043, 95% CI: 0.858, 1.267) in participants aged 65 years and over, which was similar to participants aged under 65 years (34.1% vs. 35.5%, *p* = 0.673). Furthermore, our findings revealed that a positive attitude toward the booster and recommendations from healthcare providers and family members were positively associated with vaccine willingness. Conversely, perceptions of negative impacts on cancer control and vaccine accessibility regarding the second COVID-19 booster were inversely related to the outcome event (all *p* < 0.05).

**Conclusion:**

Our study concludes with the finding of a low willingness toward the second COVID-19 booster in Chinese cancer patients, particularly in the older adults, a fact which warrants attention. This reluctance raises their risk of infection and potential for severe outcomes. Consequently, we recommend using media and community outreach to dispel misconceptions, promote the booster’s benefits, and encourage vaccine discussions with healthcare providers and family members.

## 1 Introduction

COVID-19, an infectious disease instigated by the SARS-CoV-2 virus, rapidly escalated into a worldwide pandemic since its debut in December 2019 (1). Preliminary research suggests that cancer patients and senior citizens, two key demographics, face a heightened risk of severe disease and mortality following infection with COVID-19, compared to the general population (2–4). An alarming 86.8% of COVID-19-related fatalities among 13 European nations were individuals aged 70 years and over (5). Likewise, the global prevalence of cancer in COVID-19 patients stood at 4.63%, yet a staggering 43.26% of cancer patients infected with COVID-19 suffered from severe disease manifestations (6). These figures underscore the critical need for implementing effective preventative measures, such as vaccination, to tackle the health threats posed by COVID-19 (7). Building on this, COVID-19 vaccines have demonstrated considerable efficacy in halting disease transmission, positioning themselves as one of the most cost-effective strategies to manage the pandemic (7, 8). Nonetheless, evidence points toward a gradual decrease in vaccine effectiveness against SARS-CoV-2 following the primary immunization schedule (9–11). Notably, this decline is more conspicuous among cancer patients and the older adults relative to their healthier counterparts (12–14). Contemporary evidence indicates that a second booster dose can enhance the immunogenicity of COVID-19 vaccines in these crucial groups, thereby augmenting vaccine effectiveness and reducing risks of infection, severe illness, hospitalization, and death (15, 16). To further substantiate this, the United States Food and Drug Administration (FDA) and China’s Bureau of Disease Control and Prevention endorse an additional booster dose for immunocompromised individuals, including the older adults and adults suffering from severe underlying conditions such as cancer, after completing the primary three-dose COVID-19 vaccination regimen (12, 17).

However, despite these facts, a considerable impediment to the universal willingness of the COVID-19 s booster dose is vaccine reluctance among cancer patients. Previous studies disclose that 56.4% of cancer patients display apprehension toward the second booster dose (18), attributing their concerns to potential adverse impacts on their cancer prognosis, uncertainty surrounding the vaccine’s interaction with their ongoing treatment, and fear of vaccine-related side effects (18–20). Simultaneously, in Hong Kong, China, 52.4% of the older adults displayed hesitancy toward the second booster, primarily due to worries over vaccine safety and efficacy, vaccine accessibility, perceived susceptibility to the disease, and perceived benefits of vaccination (21, 22). While prior research has probed vaccine hesitancy in both cancer patients and the older adults, the intersection of these two demographics remains a relatively unexplored territory.

Acknowledging the knowledge gap in research focused on the factors influencing vaccine hesitancy toward the COVID-19 s booster dose among older cancer patients, our study aims to understand the cognitive perceptions and willingness to vaccinate within this demographic. This understanding is crucial for health authorities to formulate targeted interventions, potentially enhancing vaccination rates in this vulnerable group. The Health Belief Model (HBM), a well-established psychological model that explains health-related behaviors. This theoretical framework allows us to explore how components of HBM, such as perceived susceptibility, benefits, barriers, and cues to action, are associated with vaccine willingness in this particular group (23).

Moreover, much of the research on vaccine willingness has predominantly been conducted in single-center settings, which restricts the representativeness and generalizability of their findings. This limitation can hinder their usefulness for understanding the factors influencing vaccination willingness for the second booster dose. Therefore, in light of these limitations, the current study utilizes a multicenter cross-sectional survey to examine the prevalence and factors influencing vaccination willingness for the COVID-19 s booster dose among older and non-older adults with cancer. By doing so, this research aspires to provide a solid scientific basis for the development of effective vaccination strategies to enhance vaccination rates among these vulnerable groups.

## 2 Materials and methods

### 2.1 Study design

Utilizing a multi-center, cross-sectional design, this study was carried out from April to June 2022 across four prominent tertiary healthcare institutions in three distinct regions of China. These encompassed Shanxi Heping Hospital (affiliated with Changzhi Medical College), Inner Mongolia People’s Hospital in North China, the First Affiliated Hospital of Xinjiang Medical University in Northwest China, and Guangdong Peking University Shenzhen Hospital in South China. The study design employed cluster sampling, wherein each of the four regional hospitals functioned as individual units for selection.

### 2.2 Participants

**Figure 1:**
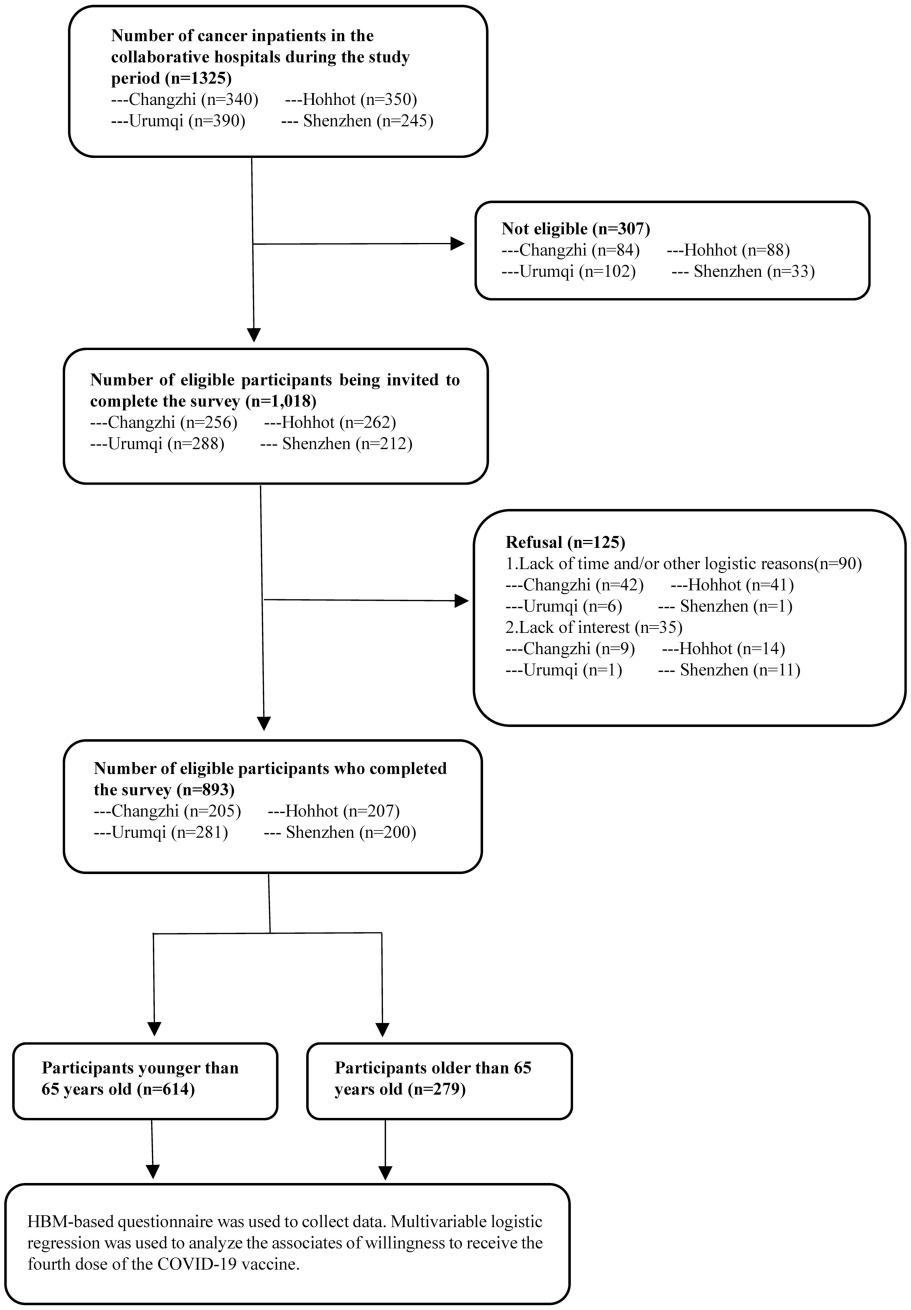
The data collection procedures of this study.

The eligibility criteria for participants included: (1) being 18 years or older; (2) being cancer patients admitted to any of the four participating hospitals during the study period; and (3) displaying a willingness to participate in the study by providing signed informed consent. Exclusions were made for individuals diagnosed with lymphoma, leukemia, or mental illness, those under medication for mental disorders, and patients having communication difficulties with researchers (Figure 1).

### 2.3 Data collection

Data was collected by medical staff from each participating hospital who were tasked with patient recruitment and screening within their respective oncology departments. Patients meeting the specified inclusion and exclusion criteria were selected via field interviewer questionnaire surveys. After acquiring informed consent, these participants completed self-administered questionnaires on the Jinshuju platform. All procedures strictly adhered to the Declaration of Helsinki’s principles, with ethical approval granted by the Institutional Review Board of Changzhi Medical College. The questionnaire-based surveys commenced only after receiving participants’ written informed consent.

### 2.4 Methods

#### 2.4.1 Design and content of the questionnaire

A team of epidemiologists, statisticians, behavioral health experts, health psychologists, and oncologists collaboratively crafted the survey questionnaire. The reliability and validity of the questionnaire were ensured via a pilot study. The individual-level variables segment of the survey drew upon the Health Belief Model (HBM), a recognized framework for studying vaccination beliefs and intentions. The survey was divided into categories covering: (1) demographic and background characteristics; (2) receipt of the first COVID-19 vaccine booster; (3) willingness to receive the second booster; (4) individual-level variables such as attitudes and perceptions toward COVID-19 vaccine boosters; (5) comprehension of the booster vaccination’s potential impact on cancer treatment; (6) vaccination fatigue; and (7) frequency of contemplating the validity of specific pandemic information. The main outcome measure was the participants’ willingness to receive the second booster dose of the COVID-19 vaccine, measured on a Likert scale (24), spanning from “strongly disagree” to “strongly agree.” For analysis purposes, responses were subsequently categorized into “unwilling to vaccinate” or “uncertain about vaccination” groups as vaccine hesitancy, following the approach adopted by Kimberly A Fisher (25).

#### 2.4.2 Sample size calculation

The sample size was calculated using a simple random sampling formula with a significance level α of 0.05, yielding Z1-α/2 = 1.96, and an allowable error δ of 0.05. We anticipated that the estimated booster dose rate for COVID-19 vaccines among cancer patients aged 65 years and over would be around 50%. The design effect (deff) was used for further sample size computation, considering the cluster sampling strategy. As per prior research (26), the deff value was set at 1.5. The initial sample size of 576, when accounting for a non-response rate of 20%, necessitated a minimum sample size of 720 respondents.

#### 2.4.3 Statistical analysis

We used descriptive statistics, the χ2 test, and Fisher’s exact test to probe associations between study outcomes and various explanatory variables. The treatment of missing data was as follows: variables with more than 30% missing data were omitted from the analysis, while for variables with less than 30% missing data, imputation techniques such as autoregressive modeling or mean imputation were applied. Autoregressive modeling is used for time-related missing data patterns, while mean imputation is used for randomly missing data.

To assess the impact of various factors on the intention to receive the second COVID-19 vaccine booster among older adults with cancer, our initial analysis involved univariate logistic regressions. These analyses evaluated the associations between demographic characteristics, specifically age groups, and booster uptake intentions. Participants were divided into two age groups: those aged 18–64 years and those aged 65 years and over. This stratification was in response to the revised focus on older adults with cancer. Significant variables (*p* < 0.1) from the univariate analysis were then included in a comprehensive multivariable logistic regression model. This model was tailored to assess the combined effect of all pertinent variables on the willingness to receive the booster dose in these distinct age groups. The approach facilitated a nuanced understanding of how each factor, particularly age, contributed to the decision-making process regarding vaccine uptake. Adjusted odds ratios (AORs) from the multivariable logistic regression provided insights into the relative importance and interplay of these variables in determining the willingness to receive the second booster dose among cancer patients, segmented by the two age groups. All data analyses were performed using IBM SPSS software (Version 25.0; IBM Corporation, Armonk, New York, USA), and statistical significance was determined using a two-sided *p*-value of less than 0.05.

## 3 Results

In this section, we present a summarized view of our key findings. Initially, we explore the background characteristics of the cancer inpatients, comparing demographic differences between age groups. Following this, we analyze factors influencing the willingness for the second COVID-19 booster dose, utilizing the Health Belief Model (HBM) as a framework. The focus will be on contrasting these factors among patients aged 65 years and over with those under 65 years.

### 3.1 Background characteristics

This section provides a detailed analysis of the demographic characteristics of the cancer patients in our study, as shown in Table 1.

**Table 1:** Background characteristics of participants.

Characteristics	> = 65 years	<65 Years	OR (95%CI)	*p*-value
	*N* (%)	*N* (%)		
Sociodemographic characteristics				
Gender				
Male	176 (63.1%)	310 (50.5%)	Reference	
Female	103 (36.9%)	304 (49.5%)	0.597 (0.447, 0.798)	<0.001
Ethnicity				
Han majority	241 (86.4%)	502 (81.8%)	Reference	
Other ethnic minorities	38 (13.6%)	112 (18.2%)	0.707 (0.474, 1.053)	0.087
Education level				
Junior high or below	208 (74.6%)	391 (63.7%)	Reference	
Senior high or equivalent	49 (17.6%)	117 (19.1%)	0.787 (0.542, 1.144)	0.209
College and above	22 (7.9%)	106 (17.3%)	0.390 (0.239, 0.636)	<0.001
Relationship status				
Single/divorced/widowed	28 (10.0%)	49 (8.0%)	Reference	
Married	251 (90.0%)	565 (92.0%)	0.777 (0.477, 1.266)	0.310
Employment status				
Full-time	39 (14.0%)	141 (23.3%)	Reference	
Part-time/self-employed/unemployed/retired/students	240 (86.0%)	465 (76.7%)	1.866 (1.267, 2.749)	0.001
Cancer related characteristics				
Type of cancer				
Lung cancer	66 (23.7%)	160 (26.1%)	0.879 (0.632, 1.223)	0.444
Gastric cancer	56 (20.1%)	51 (8.3%)	2.772 (1.840, 4.177)	<0.001
Liver cancer	11 (3.9%)	15 (2.4%)	1.639 (0.743, 3.616)	0.217
Breast cancer	7 (2.5%)	80 (13.0%)	0.172 (0.078, 0.377)	<0.001
Colorectal cancer	38 (13.6%)	113 (18.4%)	0.699 (0.469, 1.041)	0.077
Ovarian cancer	19 (6.8%)	60 (9.8%)	0.675 (0.395, 1.154)	0.149
Other cancers	134 (48.0%)	208 (33.9%)	1.804 (1.352, 2.407)	<0.001
Current treatment for cancer				
Not yet started treatment	9 (3.2%)	18 (2.9%)	Reference	
Chemotherapy only	186 (66.7%)	430 (70.0%)	0.865 (0.382, 1.961)	0.729
Radiotherapy only	37 (13.3%)	78 (12.7%)	0.949 (0.389, 2.312)	0.908
Immunotherapy only	8 (2.9%)	15 (2.4%)	1.067 (0.330, 3.448)	0.914
Others^a^	39 (14.0%)	73 (11.9%)	1.068 (0.439, 2.601)	0.884
Presence of chronic disease conditions				
No	115 (41.2%)	301 (49.0%)	Reference	
Yes	164 (58.8%)	313 (51.0%)	1.371 (1.030, 1.826)	0.030
Diabetes mellitus	18 (6.5%)	25 (4.1%)	1.625 (0.871, 3.030)	0.124
Hypertension and/or hyperlipidemia	46 (16.5%)	62 (10.1%)	1.758 (1.165, 2.651)	0.007
Chronic cardiovascular diseases	19 (6.8%)	9 (1.5%)	4.912 (2.193, 11.002)	<0.001
Chronic respiratory diseases	3 (1.1%)	2 (0.3%)	3.326 (0.553, 20.018)	0.164
Chronic liver and/or kidney diseases	6 (2.2%)	5 (0.8%)	2.677 (0.810, 8.847)	0.093
Other chronic diseases	4 (1.4%)	17 (2.8%)	0.511 (0.170, 1.532)	0.222

OR, odds ratios; CI, confidence interval; ^a^Other treatments include chemotherapy plus radiotherapy, immunotherapy plus chemotherapy or radiotherapy and completed treatment.

During the research period, 1,325 cancer inpatients were recruited from four survey sites, of which 1,018 cases (76.8%) met the inclusion criteria. After informed consent, 893 eligible participants completed the interview, among which 279 participants (31.2%) aged 65 years and over, and 614 participants (68.8%) under 65 years. Compared to the group aged less than 65, participants aged 65 years and over had a higher proportion of males (63.1% vs. 50.5%), gastric cancer (20.1% vs.8.3%), other cancers (48.0% vs. 33.9%), hypertension and hyperglycemia (16.5% vs. 10.1%) and chronic cardiovascular diseases (6.8% vs. 1.5%) (all *p* < 0.05). In contrast, older participants had a lower proportion with college or above education (7.9% vs. 17.3%), full-time employment (14.0% vs. 23.3%) and breast cancer (2.5% vs. 13.0%) compared to the younger group (all *p* < 0.05). Other characteristics did not show significant statistical differences between the two groups (*p* > 0.05) (Table 1).

### 3.2 HBM relevant variables to the willingness for the second dose of COVID-19 booster vaccination

This section analyzes how individual-level factors, based on the Health Belief Model, are connected to the willingness for the second dose of the COVID-19 booster vaccine, as shown in Table 2.

**Table 2:** The HBM relevant variables for the willingness to receive the second dose of COVID-19 booster vaccination.

	> = 65 years	<65 years	OR (95%CI)	*p*-Value
	*N* (%)	*N* (%)		
Individual-level variables				
Positive attitudes toward COVID-19 vaccine booster dose, *n* (%) agree/strongly agree				
Receiving a booster dose can maintain your antibody level and strengthen the protection against COVID-19	147 (52.7%)	331 (53.9%)	0.952 (0.717, 1.264)	0.735
A booster dose is highly effective in protecting you from COVID-19 variants of concern (e.g., Omicron)	114 (40.9%)	282 (45.9%)	0.813 (0.611, 1.083)	0.158
A booster dose is highly effective in preventing severe consequences of COVID-19	134 (48.0%)	297 (48.4%)	0.986 (0.743, 1.309)	0.924
Negative attitudes toward COVID-19 vaccine booster dose, *n* (%) agree/strongly agree				
The protection offered by COVID-19 vaccine booster dose is weaker among people with cancers	52 (18.9%)	117 (19.2%)	0.981 (0.682, 1.410)	0.916
Cancer therapy would reduce the protection of COVID-19 vaccine booster dose	98 (35.1%)	211 (34.4%)	1.034 (0.768, 1.392)	0.825
The side effects of COVID-19 vaccine booster dose are more severe among people with cancers	126 (45.2%)	260 (42.4%)	1.121 (0.843, 1.491)	0.431
The duration of protection of COVID-19 vaccine booster dose is shorter among people with cancers	95 (34.0%)	213 (34.7%)	0.972 (0.721, 1.310)	0.852
COVID-19 vaccine booster dose would negatively affect the control of cancers	118 (42.3%)	262 (42.7%)	0.985 (0.740, 1.311)	0.916
Perceived subjective norm related to COVID-19 vaccine booster dose, *n* (%) agree/strongly agree				
People who are important to you (e.g., family member, doctors) would support you to receive a booster dose	145 (52.0%)	282 (45.9%)	1.274 (0.959, 1.691)	0.094
Doctors would support you to uptake	57 (20.4%)	138 (22.5%)	0.886 (0.626, 1.253)	0.493
a booster dose				
Family member would support you to uptake a booster dose	69 (24.7%)	174 (28.3%)	0.831 (0.601, 1.148)	0.262
Perceived behavioral control to receive a COVID-19 vaccine booster dose, *n* (%) agree/strongly agree				
Receiving a COVID-19 vaccine booster dose is easy for you if you want to	128 (45.9%)	287 (46.7%)	0.966 (0.727, 1.283)	0.810
Vaccination fatigue, *n* (%) agree/strongly agree				
You are tired of receiving COVID-19 vaccination over and over again	55 (19.7%)	95 (15.5%)	1.341 (0.929, 1.937)	0.116
Media influences related to COVID-19 and vaccination				
Frequency of exposure to the following contents on TV, radio, newspaper and internet, *n* (%) sometimes/always				
Infectivity of the COVID-19	217 (77.8%)	510 (83.1%)	0.714 (0.502, 1.015)	0.060
Risk of having severe consequences or death is relatively low following infection of the COVID-19	180 (64.5%)	421 (68.6%)	0.834 (0.618, 1.124)	0.232
COVID-19 pandemic is not under control after COVID-19 vaccination rollout	152 (54.48%)	319 (51.95%)	1.107 (0.833, 1.470)	0.483
Some people become infected with COVID-19 after completion of their primary vaccine series	180 (64.52%)	375 (61.07%)	1.159 (0.864, 1.555)	0.326
Frequency of thoughtful consideration about veracity of COVID-19-specific information	149 (53.4%)	354 (57.7%)	0.842 (0.633, 1.119)	0.235

OR, odds ratios; CI, confidence interval.

There were also no significant differences in individual-level variables concerning the COVID-19 vaccine booster dose, such as positive attitudes, negative attitudes, perceived subjective norm, vaccination fatigue, consideration about the veracity of COVID-19 specific information and the Media influences related to COVID-19 and vaccination (*p* > 0.05) (Table 2).

### 3.3 Willingness for the second dose of COVID-19 booster vaccination and associated factors among patients aged 65 years and over

In this section, we examine the factors that affect the willingness of patients aged 65 years and over to get the second dose of the COVID-19 booster vaccine. Detailed results can be found in Table 3.

**Table 3:** Willingness for the second dose of COVID-19 booster vaccination and associated factors among patients aged 65 years and over (*N* = 279).

Characteristics	Prevalence of inclination n/N (%)	cOR (95%CI)	*p*-value	aOR (95%CI)	*p*-value
Individual-level variables Positive Attitude Scale					
Receiving a booster dose can maintain your antibody level and strengthen the protection against COVID-19					
No (Strongly disagree or disagree or neutral)	27/132 (20.5%)	Reference		Reference	
Yes (Strongly agree or agree)	68/147 (46.3%)	3.347 (1.965, 5.704)	<0.001	3.247 (1.901, 5.547)	**<0.001**
A booster dose is highly effective in protecting you from COVID-19 variants of concern (e.g., Omicron)					
No (Strongly disagree or disagree or neutral)	31/165 (18.8%)	Reference		Reference	
Yes (Strongly agree or agree)	64/114 (56.1%)	5.533 (3.231, 9.475)	<0.001	5.409 (3.152, 9.282)	**<0.001**
A booster dose is highly effective in preventing severe consequences of COVID-19					
No (Strongly disagree or disagree or neutral)	30/145 (20.7%)	Reference		Reference	
Yes (Strongly agree or agree)	65/134 (48.5%)	3.611 (2.135, 6.108)	<0.001	3.512 (2.071, 5.956)	**<0.001**
Negative Attitude Scale					
The protection offered by COVID-19 vaccine booster dose is weaker among people with cancers					
No (Strongly disagree or disagree or neutral)	164/492 (33.3%)	Reference		Reference	
Yes (Strongly agree or agree)	54/117 (46.2%)	2.232 (1.209, 4,121)	0.009	2.074 (1.113, 3.868)	**0.022**
Cancer therapy would reduce the protection of COVID-19 vaccine booster dose					
No (Strongly disagree or disagree or neutral)	60/181 (33.1%)	Reference		Reference	
Yes (Strongly agree or agree)	35/98 (35.7%)	1.120 (0.669, 1.877)	0.666	1.152 (0.684, 1.938)	0.595
The side effects of COVID-19 vaccine booster dose are more severe among people with cancers					
No (Strongly disagree or disagree or neutral)	58/153 (37.9%)	Reference		Reference	
Yes (Strongly agree or agree)	218/614 (35.5%)	0.681 (0.412, 1.127)	0.134	0.705 (0.425, 1.172)	0.178
The duration of protection of COVID-19 vaccine booster dose is shorter among people with cancers					
No (Strongly disagree or disagree or neutral)	63/184 (34.2%)	Reference		Reference	
Yes (Strongly agree or agree)	32/95 (33.7%)	0.976 (0.578, 1.646)	0.926	0.975 (0.576, 1.650)	0.924
COVID-19 vaccine booster dose would negatively affect the control of cancer					
No (Strongly disagree or disagree or neutral)	143/352 (40.6%)	Reference		Reference	
Yes (Strongly agree or agree)	75/262 (28.6%)	0.406 (0.239, 0.690)	0.01	0.410 (0.240, 0.699)	**0.001**
Perceived subjective norm					
People who are important to you (e.g., family member, doctors) would support you to receive a booster dose					
No (Strongly disagree or disagree or neutral)	49/134 (36.6%)	Reference		Reference	
Yes (Strongly agree or agree)	46/145 (31.7%)	0.806 (0.491, 1.323)	0.394	0.810 (0.492, 1.334)	0.407
Doctors would support you to uptake a booster					
No (Strongly disagree or disagree or neutral)	58/222 (26.1%)	Reference		Reference	
Yes (Strongly agree or agree)	37/57 (64.9%)	5.231 (2.811, 9.733)	<0.001	5.019 (2.686, 9.379)	**<0.001**
Family member would support you to uptake a booster					
No (Strongly disagree or disagree or neutral)	53/210 (25.2%)	Reference		Reference	
Yes (Strongly agree or agree)	42/69 (60.9%)	4.608 (2.593, 8.189)	<0.001	4.544 (2.549, 8.099)	**<0.001**
Perceived behavioral control					
Receiving a COVID-19 vaccine booster dose is easy for you if you want to					
No (Strongly disagree or disagree or neutral)	59/151 (39.1%)	Reference		Reference	
Yes (Strongly agree or agree)	36/128 (28.1%)	0.610 (0.368, 1.011)	0.054	0.596 (0.358, 0.992)	**0.047**
Vaccination fatigue (tired of receiving COVID-19 vaccination over and over again)					
No (Very unlikely or unlikely or neutral)	77/224 (34.4%)	Reference		Reference	
Yes (Very likely or likely)	18/55 (32.7%)	0.929 (0.496, 1.739)	0.817	0.920 (0.490, 1.730)	0.797
Interpersonal variables Media Exposure Scale					
Infectivity of the Omicron variant of COVID-19					
Almost never/seldom	17/62 (27.4%)	Reference		Reference	
Sometimes/always	78/217 (35.9%)	1.485 (0.797, 2.770)	0.212	1.462 (0.781, 2.734)	0.235
Risk of having severe consequences or death is relatively low following infection of the COVID-19					
Almost never/seldom	23/99 (23.2%)	Reference		Reference	
Sometimes/always	72/180 (40.0%)	2.203 (1.266, 3.832)	0.005	2.125 (1.217, 3.709)	**0.008**
COVID-19 pandemic is not under control after COVID-19 vaccination rollout					
Almost never/seldom	36/127 (28.3%)	Reference		Reference	
Sometimes/always	59/152 (38.8%)	1.604 (0.968, 2.658)	0.066	1.511 (0.905, 2.523)	0.114
Some people contract COVID-19 after completion of their primary vaccine series					
Almost never/seldom	31/99 (31.3%)	Reference		Reference	
Sometimes/always	64/180 (35.6%)	1.210 (0.717, 2.042)	0.474	1.121 (0.658, 1.910)	0.674
Frequency of thoughtful consideration about veracity of COVID-19-specific information					
Almost never/seldom	28/130 (21.5%)	Reference		Reference	
Sometimes/always	67/149 (45.0%)	2.976 (1.755, 5.048)	<0.001	2.824 (1.646, 4.843)	**<0.001**

Statistically significant values are identified in boldface (*p* < 0.05); cOR, crude odds ratios; aOR, Adjusted odds ratios, odds ratios adjusted for significant sociodemographic characteristics listed in Table 4 and incorporated together into the same model; CI, confidence interval.

Patients aged 65 years and over demonstrated a higher initial COVID-19 booster vaccination uptake proportion than those under 65 years (76.3% vs. 68.1%, *p* = 0.012), yet there was no significant difference shown in the willingness for a second booster among these two cancer patient groups (34.1% vs. 35.5%, *p* > 0.05). However, significant differences in the willingness for a second booster of the vaccine were found at the four survey sites (Shanxi site: 22.7%, Inner Mongolia site: 15.7%, Xinjiang site: 53.7%, Guangdong: 12.5%, *p* < 0.05).

Univariate analysis revealed a positive association between patients aged over 65 years from other ethnic minorities and the willingness for the fourth COVID-19 vaccine booster vaccination (Table 4).

**Table 4:** Associations between background characteristics and the willingness of the second COVID-19 vaccine booster vaccination (*N* = 893).

	Cancer patients aged over 65 years (*N* = 279)			Cancer patients aged <65 years (*N* = 614)		
	Prevalence of inclination n/N (%) (279)	OR (95%CI)	*p*-values	Prevalence of inclination n/N (%) (614)	OR (95%CI)	*p*-values
Gender						
Male	60/176 (34.1%)	Reference		134/310 (43.2%)	Reference	
Female	35/103 (34.0%)	0.995 (0.596, 1.662)	0.985	84/304 (27.6%)	0.501 (0.358, 0.703)	**<0.001**
Ethnicity						
Han majority	77/241 (32.0%)	Reference		159/502 (31.7%)	Reference	
Other ethnic minorities	18/38 (47.4%)	1.917 (0.960, 3.829)	**0.062**	59/112 (52.7%)	2.401 (1.584, 3.640)	**<0.001**
Education level						
Junior high or below	66/208 (31.7%)	Reference		124/391 (31.7%)	Reference	
Senior high or equivalent	20/49 (40.8%)	1.484 (0.782, 2.814)	0.227	43/117 (36.8%)	1.251 (0.812, 1.927)	0.309
College and above	9/22 (40.8%)	1.490 (0.606, 3.659)	0.385	51/106 (48.1%)	1.997 (1.290, 3.089)	**0.002**
Relationship status						
Single/divorced/widowed	8/28 (28.6%)	Reference		18/49 (36.7%)	Reference	
Married	87/251 (34.7%)	1.326 (0.561, 3.134)	0.851	200/565 (35.4%)	0.944 (0.515, 1.730)	0.519
Employment status						
Full-time	15/39 (38.5%)	Reference		50/141 (35.5%)	Reference	
Part-time/self-employed/unemployed/retired/students	80/240 (33.3%)	0.800 (0.398, 1.609)	0.531	161/465 (34.6%)	0.964 (0.650, 1.430)	0.855
Type of cancer						
Lung cancer	22/66 (33.3%)	0.959 (0.810, 1.200)	0.888	70/160 (43.8%)	1.608 (1.112, 2.326)	**0.011**
Gastric cancer	16/56 (28.6%)	0.729 (0.384, 1.385)	0.333	21/51 (41.2%)	1.301 (0.725, 2.332)	0.377
Liver cancer	2/11 (18.2%)	0.418 (0.089, 1.975)	0.257	4/15 (26.7%)	0.654 (0.206, 2.080)	0.469
Breast cancer	1/7 (14.3%)	0.316 (0.037, 2.660)	0.264	9/80 (11.3%)	0.197 (0.096, 0.403)	**<0.001**
Colorectal cancer	12/38 (31.6%)	1.331 (0.779, 2.274)	0.294	30/113 (26.5%)	1.451 (0.876, 2.405)	0.147
Esophageal cancer	8/15 (53.3%)	2.325 (0.817, 6.620)	0.105	12/25 (48.0%)	1.716 (0.769, 3.830)	0.183
Ovarian cancer	9/19 (47.4%)	1.821 (0.714, 4.647)	0.204	18/60 (30.0%)	0.759 (0.425, 1.353)	0.348
Other cancers	29/74 (39.2%)	1.627 (1.037, 2.554)	0.276	77/156 (49.4%)	2.023 (1.282, 3.193)	**<0.001**
Current treatment for cancer						
Not yet started treatment	2/9 (22.2%)	Reference		6/18 (33.3%)	Reference	
Chemotherapy only	60/186 (32.3%)	1.667 (0.336, 8.265)	0.532	118/430 (27.4%)	0.756 (0.278, 2.062)	0.756
Radiotherapy only	17/37 (45.9%)	2.975 (0.544, 16.273)	0.209	54/78 (69.2%)	4.500 (1.511, 13,405)	**0.007**
Immunotherapy only	1/8 (12.5%)	0.500 (0.036, 6.862)	0.604	7/15 (46.7%)	1.740 (0.427, 7.171)	0.437
^a^Others	15/39 (38.5%)	2.188 (0.400, 11.959)	0.366	33/73 (45.2%)	1.650 (0.559, 4.873)	0.365
Presence of any other chronic diseases						
No	35/115 (30.4%)	Reference		111/301 (36.9%)	Reference	
Yes	60/164 (36.6%)	1.319 (0.793, 2.193)	0.286	107/313 (34.2%)	0.889 (0.639, 1.238)	0.486

Statistically significant values are identified in boldface (*p* < 0.1); OR: odds ratios; CI: confidence interval; ^a^Other treatments include chemotherapy plus radiotherapy, immunotherapy plus chemotherapy or radiotherapy and completed treatment.

The multivariable logistic regression was used to adjust for significant background variables (*p* < 0.1) and to incorporate them into the same model. Post adjustment for the ethnic minorities factor, several variables, such as positive attitudes about the vaccine’s effectiveness in maintaining antibody levels (AOR: 3.247, 95% CI: 1.901, 5.547), protection against COVID-19 variants (AOR: 5.409, 95% CI: 3.152, 9.282), and prevention of severe COVID-19 consequences (AOR: 3.512, 95% CI: 2.071, 5.956), correlated with a higher willingness for the booster dose. Negative attitudes about the booster’s efficacy in cancer patients also influenced the inclination for the booster dose, such as the protection of booster dose is weaker among cancers (AOR: 2.074, 95% CI: 1.113, 3.868) while control of cancers is negatively affected among cancer patients (AOR: 0.410, 95% CI: 0.240, 0.699) (Table 3).

Additionally, the support of doctors and family member promoted higher uptake willingness of booster dose (AOR: 5.019, 95% CI: 2.686, 9.379 and AOR: 4.544, 95% CI: 2.549, 8.099). However, perceived behavioral control such as accessibility of vaccines hindered the uptake willingness of the booster dose (AOR: 0.596, 95% CI: 0.358, 0.992). Furthermore, the media exposure scale about the low fatality and severity of variations and frequent consideration about the veracity of COVID-19 specific information significantly promoted higher willingness uptake of the booster dose (AOR: 2.125, 95% CI: 1.217, 3.709 and AOR: 2.824, 95% CI: 1.646, 4.843) (Table 3).

### 3.4 Willingness for the second dose of COVID-19 booster vaccination and associated factors among patients under 65 years.

In this section, we examine the factors that affect the willingness of patients under 65 years to get the second dose of the COVID-19 booster vaccine. Detailed results can be found in Table 5.

**Table 5:** Willingness for the second dose of COVID-19 Booster vaccination and associated factors among patients under 65 years (*N* = 614).

Characteristics	Prevalence of inclination n/N (%)	cOR (95%CI)	*p*-value	aOR (95%CI)	*p*-value
Individual-level variables Positive Attitude Scale					
Receiving a booster dose can maintain your antibody level and strengthen the protection against COVID-19					
No (Strongly disagree or disagree or neutral)	53/283 (18.7%)	Reference		Reference	
Yes (Strongly agree or agree)	165/331 (49.8%)	4.313 (2.985, 6.234)	<0.001	4.805 (3.179, 7.262)	**<0.001**
A booster dose is highly effective in protecting you from COVID-19 variants of concern (e.g., Omicron)					
No (Strongly disagree or disagree or neutral)	62/332 (18.7%)	Reference		Reference	
Yes (Strongly agree or agree)	156/282 (55.3%)	5.392 (3.753, 7.746)	<0.001	4.915 (3.320, 7.277)	**<0.001**
A booster dose is highly effective in preventing severe consequences of COVID-19					
No (Strongly disagree or disagree or neutral)	62/317 (19.6%)	Reference		Reference	
Yes (Strongly agree or agree)	156/297 (52.5%)	4.550 (3.178, 6.516)	<0.001	4.202 (2.844, 6.208)	**<0.001**
Negative Attitude Scale					
The protection offered by COVID-19 vaccine booster dose is weaker among people with cancers					
No (Strongly disagree or disagree or neutral)	69/223 (30.9%)	Reference		Reference	
Yes (Strongly agree or agree)	26/52 (50.0%)	1.714 (1.139, 2.580)	0.009	1.739 (1.103, 2.741)	**0.017**
Cancer therapy would reduce the protection of COVID-19 vaccine booster dose					
No (Strongly disagree or disagree or neutral)	149/403 (37.0%)	Reference		Reference	
Yes (Strongly agree or agree)	69/211 (32.7)	0.828 (0.583, 1.177)	0.294	0.884 (0.602, 1.299)	0.531
The side effects of COVID-19 vaccine booster dose are more severe among people with cancers					
No (Strongly disagree or disagree or neutral)	137/354 (38.7%)	Reference		Reference	
Yes (Strongly agree or agree)	81/260 (31.2%)	0.717 (0.511, 1.006)	0.053	0.841 (0.580, 1.219)	0.361
The duration of protection of COVID-19 vaccine booster dose is shorter among people with cancers					
No (Strongly disagree or disagree or neutral)	147/401 (36.7%)	Reference		Reference	
Yes (Strongly agree or agree)	71/213 (33.3%)	0.864 (0.609, 1.226)	0.412	0.972 (0.662, 1.427)	0.884
COVID-19 vaccine booster dose would negatively affect the control of cancer					
No (Strongly disagree or disagree or neutral)	68/161 (42.2%)	Reference		Reference	
Yes (Strongly agree or agree)	27/118 (22.9%)	0.586 (0.416, 0.825)	0.02	0.711 (0.489, 1.033)	0.074
Perceived subjective norm					
People who are important to you (e.g., family member, doctors) would support you to receive a booster dose					
No (Strongly disagree or disagree or neutral)	122/332 (36.7%)	Reference		Reference	
Yes (Strongly agree or agree)	96/282 (34.0%)	0.888 (0.637, 1.239)	0.485	0.779 (0.540, 1.125)	0.183
Doctors would support you to uptake a booster					
No (Strongly disagree or disagree or neutral)	130/476 (27.3%)	Reference		Reference	
Yes (Strongly agree or agree)	88/138 (63.8%)	4.684 (3.136, 6.998)	<0.001	3.794 (2.432, 5.918)	**<0.001**
Family member would support you to uptake a booster					
No (Strongly disagree or disagree or neutral)	113/440 (25.7%)	Reference		Reference	
Yes (Strongly agree or agree)	105/174 (60.3%)	4.404 (3.037, 6.385)	<0.001	4.237 (2.798, 6.416)	**<0.001**
Perceived behavioral control					
Receiving a COVID-19 vaccine booster dose is easy for you if you want to					
No (Strongly disagree or disagree or neutral)	125/327 (38.2%)	Reference		Reference	
Yes (Strongly agree or agree)	93/287 (32.4%)	0.775 (0.555, 1.081)	0.133	0.626 (0.432, 0.908)	**0.014**
Vaccination fatigue (tired of receiving COVID-19 vaccination over and over again) 133					
No (Very unlikely or unlikely or neutral)	180/519 (34.7%)	Reference		Reference	
Yes (Very likely or likely)	38/95 (40.0%)	1.256 (0.802, 1.966)	0.319	1.099 (0.671, 1.800)	0.707
Interpersonal variables Media Exposure Scale					
Infectivity of the Omicron variant of COVID-19					
Almost never/seldom	25/104 (24.0%)	Reference		Reference	
Sometimes/always	193/510 (37.8%)	1.924 (1.186, 3.122)	0.007	1.619 (0.956, 2.743)	0.073
Risk of having severe consequences or death is relatively low following infection of the COVID-19					
Almost never/seldom	53/193 (27.5%)	Reference		Reference	
Sometimes/always	165/421 (39.2%)	1.703 (1.174, 2.469)	0.005	1.470 (0.979, 2.205)	0.063
COVID-19 pandemic is not under control after COVID-19 vaccination rollout					
Almost never/seldom	91/295 (30.8%)	Reference		Reference	
Sometimes/always	127/319 (39.8%)	1.483 (1.062, 2.070)	0.020	1.219 (0.844, 1.760)	0.291
Some people contract COVID-19 after completion of their primary vaccine series					
Almost never/seldom	76/239 (31.8%)	Reference		Reference	
Sometimes/always	142/375 (37.9%)	1.307 (0.928, 1.842)	0.126	1.041 (0.710, 1.526)	0.836
Frequency of thoughtful consideration about veracity of COVID-19-specific information					
Almost never/seldom	42/260 (16.2%)	Reference		Reference	
Sometimes/always	176/354 (49.7%)	5.132 (3.473, 7.584)	<0.001	4.153 (2.716, 6.352)	**<0.001**

Statistically significant values are identified in boldface (*p* < 0.05); cOR, crude odds ratios; aOR, adjusted odds ratios, odds ratios adjusted for significant sociodemographic characteristics listed in Table 4 and incorporated together into the same model; CI, confidence interval.

In the under 65 years group, certain demographic factors, including other ethnicities, having a college or higher education, specific types of cancer, and current treatment regimen, positively associated with the willingness for the booster. Conversely, being female and having breast cancer negatively associated with the propensity for the booster (Table 4).

The multivariable logistic regression, adjusted for these significant factors (*p* < 0.1) and to incorporate them into the same model, revealed similar associations as seen in the older age group, including attitudes about the vaccine’s effectiveness, the support of doctors and family member (AOR: 5.019, 95% CI: 2.686, 9.379 and AOR: 4.544, 95% CI: 2.549, 8.099), and frequent consideration about the veracity of COVID-19 specific information (AOR: 4.153, 95% CI: 2.716, 6.352). Vaccine accessibility negatively correlated with the propensity for the second booster dose (AOR: 0.626, 95% CI: 0.432, 0.908) (Table 5).

## 4 Discussion

Our multi-center cross-sectional study examined the levels of vaccine hesitancy and its related factors among older adults with cancer in China concerning the second COVID-19 booster dose. Our findings revealed a markedly low inclination to receive the second booster, with only one-third of Chinese older adults with cancer expressing such a willingness. Key determinants of this hesitancy included the perceived COVID-19 threat, perceived benefits, self-efficacy, and cues to action. This study not only fills a gap in existing research but also lays the foundation for the development of targeted strategies to mitigate hesitancy and boost vaccination coverage rates within this highly vulnerable population.

Interestingly, we found no significant difference in the willingness to receive the second booster between older and non-older adults with cancer (34.1% vs. 35.5%, *p* = 0.673). This finding underscores the importance of addressing vaccination hesitancy across all age groups among cancer patients, as both these groups demonstrated lower willingness compared to previous studies in Hong Kong, China, and the United States (22, 27). However, given that cancer patients over 65 years have a higher risk of COVID-19 infection and death than younger cancer patients, more attention should be paid to promoting the second dose of COVID-19 vaccine for cancer patients over 65 years. However, the notable disparities in vaccine willingness observed among cancer patients residing in distinct regions necessitate targeted efforts in health education to address lower willingness rates in these areas.

Given the elevated vulnerability of both older adults and cancer patients to COVID-19 complications, it is crucial to confront hesitancy toward the second booster. According to our findings, one of the major determinants of this hesitancy was the fear that the vaccine could interfere with cancer treatment. This fear was more prevalent among older adults with cancer aged 65 years and over, and absent in the under-65 years group. This may be due to the fact that older adults with cancer over 65 years are relatively inconvenient to obtain external information through the Internet, smartphone and other means. Therefore, China needs to increase publicity and education on related concerns of older adults with cancer to eliminate their doubts when carrying out the second booster injection of COVID-19 vaccine for groups with underlying diseases.

Unexpectedly, we found that both older and non-older adults with cancer who perceived the booster as ineffective exhibited a higher willingness to get vaccinated. This paradox may be linked to a heightened awareness of COVID-19’s severity, leading to vaccine skepticism yet willingness. This hypothesis, however, requires further exploration.

Contrary to our expectations, our study found that easier access to vaccination was associated with diminished willingness to vaccinate. This may be explained by the “Veblen effect” (28), whereby easily attainable commodities are perceived as less valuable. Despite this, it remains crucial to improve vaccine accessibility among cancer patients.

We discovered that the attitudes toward booster shots, as well as recommendations from healthcare providers and family members, were positively correlated with the vaccine willingness among cancer patient participants. These findings suggest that garnering support from relatives, healthcare professionals, and the broader community is pivotal in promoting the uptake of the second dose of the COVID-19 booster vaccine among Chinese cancer patients.

This study boasts two main strengths. Firstly, its design as a multi-center cross-sectional survey ensures a broader representation of respondents compared to a single-center study. Secondly, our research undertook a side-by-side comparison of the willingness of cancer patients, both over and under 65 years of age, to receive the second COVID-19 vaccine booster dose. This comparison, along with an analysis of associated factors, offers valuable insights for guiding current vaccination promotion and education efforts targeted at the Chinese population. This study does have limitations. Firstly, the survey was conducted in the specific context of China’s epidemic strategy, so patient views and behaviors could change with evolving policies. Secondly, the focus on hospitalized cancer patients in four specific prominent tertiary healthcare institutions may limit the broader applicability of our findings to all cancer patients in China. Lastly, the cross-sectional nature of our study does not allow for causal inferences. Future research should be expanded to encompass a broader sample of the population, including diverse regional environments or a more extensive group of non-hospitalized cancer patients, and adapt to continuously evolving strategies to achieve more comprehensive applicability.

In conclusion, given the pronounced lack of willingness for the second COVID-19 vaccine booster shot among cancer patients, health departments must intensify their efforts. Strategies should aim to increase understanding of the severity of COVID-19 and the benefits of vaccination, especially among 65 years and over older adults with cancer. Differentiated approaches for patients of varying ages, while ensuring vaccine accessibility, are crucial. Further exploration should focus on the vaccine’s potential impact on cancer treatment and include non-hospitalized patients.

## Data availability statement

The raw data supporting the conclusions of this article will be made available by the authors, without undue reservation.

## Ethics statement

The studies involving humans were approved by Institutional Review Board (or Ethics Committee) of Institutional Review Board of Changzhi Medical College (protocol code RT2022027). The studies were conducted in accordance with the local legislation and institutional requirements. Written informed consent for participation in this study was provided by the participants' legal guardians/next of kin.

## Author contributions

LiZ: Formal analysis, Software, Writing – original draft, Writing – review & editing. JY: Data curation, Project administration, Resources, Supervision, Writing – review & editing. RS: Data curation, Project administration, Resources, Supervision, Writing – review & editing. XL: Data curation, Supervision, Writing – review & editing. MS: Data curation, Supervision, Writing – review & editing. LeZ: Conceptualization, Methodology, Supervision, Writing – review & editing. JX: Conceptualization, Formal analysis, Funding acquisition, Methodology, Project administration, Supervision, Writing – review & editing, Writing – original draft.
